# Antipsychotic treatment experiences of people with bipolar I disorder: patient perspectives from an online survey

**DOI:** 10.1186/s12888-020-02767-x

**Published:** 2020-07-10

**Authors:** Leona Bessonova, Dawn I. Velligan, Peter J. Weiden, Amy K. O’Sullivan, Aaron Yarlas, Martha Bayliss, Nishtha Baranwal, Kaitlin Rychlec, Julia Carpenter-Conlin, Michael J. Doane, Martha Sajatovic

**Affiliations:** 1 Alkermes, Inc., 852 Winter Street, Waltham, MA USA; 2 The University of Texas Health Science Center at San Antonio, 7703 Floyd Curl Drive, San Antonio, TX USA; 3 Optum, 1301 Atwood Avenue, Johnston, RI USA; 4 University Hospitals Cleveland Medical Center, 11100 Euclid Avenue, Cleveland, OH USA

**Keywords:** Bipolar I disorder, Bipolar disorder, Antipsychotic, Side effects, Adherence, Preference

## Abstract

**Background:**

Oral antipsychotic (AP) medications are frequently prescribed to people with bipolar I disorder (BD-I). A cross-sectional online survey examined the experiences of people living with BD-I with a history of recent AP use.

**Methods:**

Adults with self-reported physician-diagnosed BD-I (*N* = 200) who received oral APs during the prior year completed a survey on AP-related experiences, including side effects and their perceived burden on social functioning, adherence, and work. Items also assessed preferences for trade-offs (balancing symptom management and side effects) when considering a hypothetical new AP. The perceived impact of specific, prevalent side effects on adherence, work, and preferences for a hypothetical AP were also examined. Analyses were descriptive.

**Results:**

The survey sample had a mean age of 43.2 (SD = 12.4) years, was 60% female, and 31% nonwhite. Almost all participants (98%) had experienced AP side effects. Common self-reported side effects were feeling drowsy or tired (83%), lack of emotion (79%), anxiety (79%), dry mouth (76%), and weight gain (76%). Weight gain was cited as the most bothersome side effect, rated by most participants (68%) as “very” or “extremely bothersome.” Nearly half of participants (49%) reported that AP side effects negatively impacted their job performance; almost all (92%) reported that side effects – most commonly anxiety and lack of emotion – negatively impacted social relationships (e.g., family or romantic partners). The most commonly-reported reason for stopping AP use was dislike of side effects (48%). Side effects most likely to lead to stopping or taking less of AP treatment included “feeling like a ‘zombie’” (29%), feeling drowsy or tired (25%), and weight gain (24%). When considering a hypothetical new AP, the most common side effects participants wanted to avoid included AP-induced anxiety (50%), weight gain (48%), and “feeling like a ‘zombie’” (47%).

**Conclusions:**

Side effects of APs were both common and bothersome, and impacted social functioning, adherence, and work. Findings highlight the prevailing unmet need for new APs with more favorable benefit-risk profiles.

## Background

Bipolar disorder (BD) includes a group of chronic mood disorders involving at least one lifetime manic or mixed-manic episode and often involves depressive episodes [1]. Bipolar I disorder (BD-I) is characterized by a lifetime history of at least one manic episode as well as depressive episodes, which last 2 weeks or longer [1].

The pharmacologic treatment of BD has evolved over the past few decades. In addition to traditional mood stabilizers such as lithium and selected anticonvulsant drugs, antipsychotics (APs) are widely used to treat BD [2, 3]. Both first-generation (FGAs) and second-generation APs (SGAs) effectively manage symptoms of BD, but are associated with a variety of side effects (e.g., extrapyramidal symptoms [EPS], metabolic syndrome, weight gain, dyslipidemia, blood pressure, and liver toxicity) [4]. It is estimated that 60 to 80% of people with BD have been treated with at least one AP during their lifetime, including FGAs or SGAs [5–7].

Previous research has investigated patient attitudes around BD treatment using mixed research methods and stated preference approaches [8, 9]. For example, patients have cited weight gain and cognitive effects as the two side effects most likely to reduce medication adherence to a hypothetical treatment [8]. In BD, non-adherence to treatment is common [10, 11]; non-adherence to APs is associated with increased risk of relapse and suicide attempts [12], as well as increased health care resource utilization, including emergency room visits and hospitalization [13]. What has been less documented includes patients’ perspectives on the bothersomeness of specific AP side effects, whether these side effects are perceived to impact social functioning and work, and preferences for balancing symptom management and side effects (i.e., benefit-risk trade-offs) in APs.

### Study objective

The primary objective of this cross-sectional web-based survey was to characterize the experiences of people with BD-I who had experience with an oral AP in the last year. The survey focused on AP side effects, side effect burden, and perceived impacts of side effects on social functioning, adherence, and work. Further, we captured patients’ preferences for trade-offs when considering a hypothetical new AP.

## Methods

### Research design

This cross-sectional, observational, web-based survey assessed experiences of people with BD-I, particularly regarding use of and experiences with APs. The protocol, informed consent form (ICF), participant screener, and survey form were approved by the New England Independent Review Board.

The target population was 200 adults with BD-I with a history of AP use in the last year (either FGA or SGA). Participants were eligible if they were at least 18 years of age, resided in the US, self-reported a clinician-diagnosis of BD-I at least 12 months prior to enrollment, self-reported ongoing treatment by a mental health care professional (MHCP) for at least 3 months prior, and self-reported oral AP use during the prior year for a minimum of 1 week. Participants were not eligible if they reported any diagnosis of schizophrenia or schizoaffective disorder, had been hospitalized for psychiatric care within the last 3 months prior, or if they participated in a clinical treatment trial for psychiatric disorders during the prior year.

### Screening procedures and data collection

Participants were identified by Schlesinger Group, a firm specializing in recruiting research participants. Schlesinger Group collaborated with three organizations, each running a pre-existing panel of individuals interested in participating in survey research assembled using in-person, phone, and online recruitment strategies. Initial membership surveys, including demographic, medical, and lifestyle information, were collected from all panel members. The panel administrators employed regular validation procedures to ensure the quality and authenticity of panel members (e.g., digital fingerprinting, flagging of suspicious IP addresses, etc.). For this survey, Schlesinger Group identified adult panel members with a self-reported diagnosis of BD (based on initial membership surveys) and invited them to participate via email. The invitation email contained a link to the secure webpage hosting the ICF.

Participants completed all survey materials online – including an ICF and a screener (that included items used to assess eligibility). Potential participants were administered the ICF electronically via a secure web-based data capture system, prior to screening, and were given as much time as they needed to consider participation. Potential participants were informed that they could decline participation without providing a reason and discontinue participation from the survey at any time without penalty. Contact information was included for both the study PI and the IRB, in case there were questions about the research. Participants indicated their consent by marking a checkbox and typing their initials. Following electronic completion of the ICF and the screener, participants who met eligibility criteria were invited to complete the full web-based survey. The survey was conducted from December 14th, 2018 to January 15th, 2019.

To optimize representativeness to the larger population of BD-I patients, enrollment quotas for age and gender were instituted [14–16]. The target distribution included a female to male ratio of 3:2 (or vice versa) so that neither gender exceeded 120 participants, and age distribution of no more than 30% of participants under age 30 and at least 20% of participants over age 50.

### Survey design

The survey included both validated instruments and items developed by investigators. The Treatment Satisfaction Questionnaire for Medication Version II (TSQMvII) [17] measures satisfaction with a specified medication over the previous 2 to 3 weeks across four domains: effectiveness, convenience, side effects, and global satisfaction. The TSQMvII was administered only to participants currently taking an oral AP medication, and items were asked about that medication. The response choices for all TSQMvII items use a 7-point Likert-type scale (“extremely dissatisfied” to “extremely satisfied”) except for items belonging to the side effects domain, which use a 5-point Likert-type scale (“extremely dissatisfied” to “not at all dissatisfied”). Scores for each domain are the average of responses adjusted to range from 0 to 100, where higher scores correspond to greater satisfaction.

The remainder of the survey consisted of items developed by investigators to capture detailed feedback about BD-I with a focus on experiences with APs (see Additional file 1). Content for these items, including symptom and side effect lists, was sourced from a targeted literature review, focus groups among patients with BD-I, which elicited insights about experiences with the disease and AP treatment [18], and input from expert clinicians, psychometricians, and representatives from national mental health advocacy groups.

Developed items captured the length of time since participants’ last manic and depressive episodes, and the symptoms they experienced during their most recent episodes. Participants were given a list of 16 symptoms of mania and 13 of depression and were also allowed to add symptoms that were not listed. Participants were also given a list of 12 common AP side effects and asked to endorse those they had experienced (i.e., anxiety, gastrointestinal problems, dizziness/fainting, dry mouth, feeling a lack of emotion, feeling drowsy or tired, “feeling like a ‘zombie,’” involuntary movements, restlessness, sexual dysfunction, trouble concentrating, weight gain). In addition to the 12 listed side effects, participants were able to spontaneously add and rate additional side effects. For side effect experienced, participants were then asked to rate its bothersomeness on a 4-point scale (not, somewhat, very, or extremely bothersome). Items addressing further aspects of AP experiences included perceived impacts of AP side effects on social functioning (interactions with other people, relationships with family and friends, romantic relationships, and feeling embarrassed in front of other people), modification of oral AP treatment regimen (with or without agreement of their MHCP^1^), and work (e.g., job performance, relationships with co-workers).

The survey also captured participants’ preferences for a hypothetical new AP. These items elicited trade-offs participants were willing to make between the efficacy of symptom management and experiencing specific side effects. Participants were asked to select up to five side effects that they most wanted to avoid in a hypothetical new oral AP from the same list of 12 common AP side effects described earlier. For each of the side effects (up to five) for which the participant had reported they most wanted to avoid in a hypothetical new oral AP, participants were asked to choose one of the following four options, that compared changes in symptoms and side effects relative to those experienced with their current or most recent oral AP medication: 1) large improvement in BD-I symptoms, but a slight worsening of that side effect; 2) small improvements in BD-I symptoms, with no change in that side effect; 3) no change in BD-I symptoms, with a small improvement in that side effect; or 4) slight worsening in BD-I symptoms, but a large improvement in that side effect.

### Analysis

Only data from participants who completed the survey were included in analyses. Responses were required for all survey items; however, due to technology problems, a small number of items were not administered to one or two participants. These participants were considered to have completed the survey; thus, their responses to all other items were included in analyses. Items developed for this survey were evaluated individually without aggregation. Data collected in open-ended items (e.g., “other” manic or depressive symptoms, or AP side effects) were tabulated when reported by three or more participants.

Univariate analyses included descriptive statistics for survey items. Categorical data are reported using frequencies and percentages, while continuous data are reported using means and standard deviations (SDs).

The statistical analysis plan (SAP) for this survey prespecified that perceived impacts would be examined for AP side effects experienced by at least half of participants with BD-I identified in a completed qualitative focus group study conducted to inform the development of this survey.^2^ In this qualitative study, only two side effects were spontaneously reported by a majority of focus group participants: weight gain (83%) and feeling drowsy or tired (63%) [18]. Bivariate analyses in the current study consisted of separate cross tabulations by the level of bothersomeness for these two side effects for frequencies related to modification of treatment regimen, work, and preferences for a hypothetical new AP. For bivariate analyses, the two highest levels of bothersomeness (i.e., very, extreme) regarding weight gain or feeling drowsy or tired were combined, as prespecified in the SAP, into a single group due to the conceptual similarity of these responses, while the two lowest level of bothersomeness (i.e., none, somewhat) were combined into a single group due to the small number of participants who selected ‘not bothersome’ for these two side effects (which was below the minimum number specified in the SAP for maintaining separate subgroups).

All analyses were descriptive; no hypothesis testing was performed. Thus, a formal power analysis was not conducted. A sample size of 200 participants was determined to be sufficient to describe the characteristics (i.e., age and gender) and experiences of people living with BD-I. All analyses were conducted using SAS 9.4 (Cary, NC: SAS Institute Inc.).

## Results

### Participant characteristics

The final sample included 200 participants (See Additional file 2 – Figure S1). As noted in Table 1, mean age was 43.2 years old (SD = 12.4); more participants were female (60%). Almost one-third of participants were non-Caucasian (31%), with 11% Hispanic/Spanish/Latino. The most common level of education completed was some college (with no degree; 30%) followed by bachelor’s degree (25%). At the time of the survey, 42% of participants worked for pay (full- or part-time) and nearly one-quarter of participants received disability payments (24%).

**Table 1:** Participant Characteristics

Characteristic	***N*** = 200
**Age in Years, mean (SD)**	43.2 (12.4)
**Female, n (%)**	119 (59.5%)
**Race, n (%)**	
African-American or Black	18 (9.0%)
American Native	4 (2.0%)
Asian	7 (3.5%)
Caucasian or White	138 (69.0%)
Hawaiian Native or other Pacific Islander	1 (0.5%)
Multiple Races	21 (10.5%)
Other	11 (4.5%)
**Ethnicity, n (%)**	
Hispanic/Spanish/Latino origin	21 (10.5%)
**Education, n (%)**	
Less than High School Diploma	4 (2.0%)
High School Diploma or GED	36 (18.0%)
Some College, but no degree	60 (30.0%)
Associate’s Degree or Technical Certificate	27 (13.5%)
Bachelor’s Degree (B.A., B.S., etc.)	49 (24.5%)
Graduate Degree (M.A., M.S., Ph.D. M.D., etc.)	24 (12.0%)
**Current Employment Status**^**a**^	
Employed (working for pay) full-time	63 (31.5%)
Disability	48 (24.0%)
Retired	22 (11.0%)
Employed (working for pay) part-time	21 (10.5%)
Homemaker	15 (7.5%)
Unemployed and looking for work	12 (6.0%)
Student	12 (6.0%)
Unemployed and not looking for work	4 (2.0%)
Other	3 (1.5%)
**Time Since BD-I Diagnosis, n (%)**	
1 year to < 2 years	19 (9.5%)
2 years to < 3 years	16 (8.0%)
3 years to < 5 years	41 (20.5%)
5 years to < 10 years	38 (19.0%)
≥ 10 years	86 (43.0%)
**Currently Taking Oral AP, n (%)**^**b**^	162 (81.4%)

*AP* Antipsychotic, *SD* Standard Deviation

^a^ Employment status categories are listed in order of descending frequency

^b^ One participant’s response was not recorded due to a technology problem, and has been excluded from frequency calculations

Most participants had been diagnosed with BD-I for longer than 3 years prior to the survey (83%), 62% more than 5 years prior, and 43% more than 10 years prior.

### BD-I episodes: commonly reported symptoms

More participants indicated that they experienced depressive episodes (67%) compared to manic episodes (51%) within the month prior to survey administration.

During their most recent depressive episodes, participants’ most frequently experienced symptoms were decreased pleasure or interest in activities (79%), low mood (76%), feeling tired or loss of energy (75%), anxiety (75%), avoiding interactions with friends, family, or co-workers (74%), decreased sleep or sleeping too much (69%), difficulty thinking, concentrating, or making decisions (61%), and feeling worthless or guilty (61%) (See Additional file 2 – Table S1).

During their most recent manic episodes, participants’ most frequently experienced symptoms were anxiety (85%), decreased sleep (77%), feeling restless (76%), racing thoughts (72%), feeling angry or irritated with friends, family, or co-workers (70%), and difficulty paying attention to one thing (70%) (See Additional file 2 – Table S2).

### Self-reported AP treatment experience

One hundred and sixty-two participants (81%) reported taking at least one AP at the time of the survey. Of these, 78% were taking one AP, 16% were taking two APs, and the remaining 6% were taking three or more APs. The five most frequently reported APs for participants in this survey (combining current and previous medications) included aripiprazole (57%), quetiapine (52%), risperidone (37%), olanzapine (29%), and lurasidone (24%). The five AP medications with the most frequent current usage were quetiapine (23%), aripiprazole (17%), lurasidone (12%), risperidone (10%), and olanzapine (10%).

TSQMvII scores indicated participants currently taking an AP rated the convenience of AP medication between “satisfied” to “very satisfied” (mean = 74.8, SD = 17.8). Lower scores were observed for effectiveness of AP medication (“somewhat satisfied” to “satisfied,” mean = 61.0, SD = 21.5) and global satisfaction of AP medication (“satisfied,” mean = 65.5, SD = 20.3). Among participants that experienced side effects of current AP medication (99 of 161 who were administered the TSQMvII), low scores on the side effects scale (mean = 61.2, SD = 23.0) were observed, which corresponded to ratings between “somewhat dissatisfied” and “slightly dissatisfied.”

### Self-reported AP side effects

#### Overall experiences

**Fig. 1:**
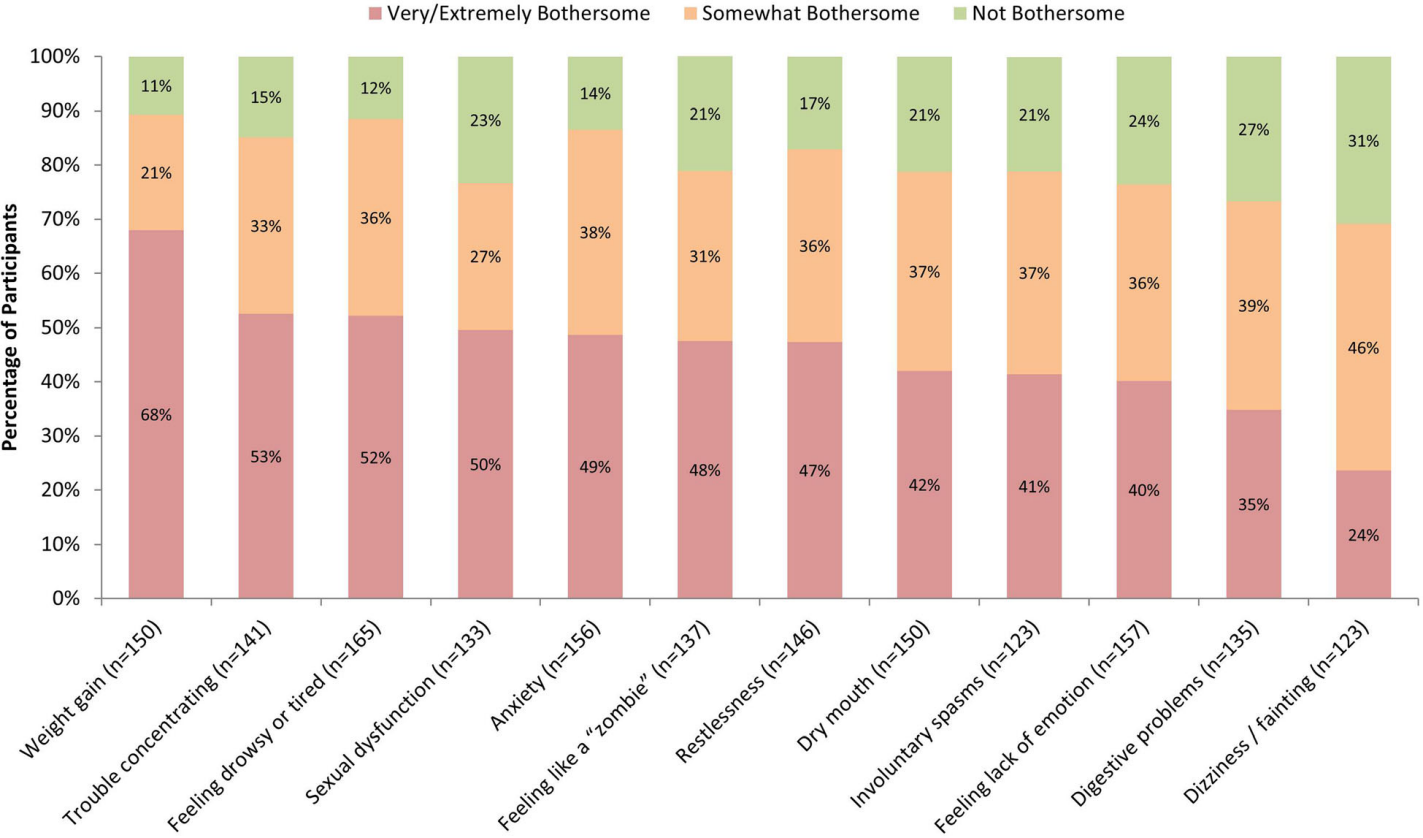
Bothersomeness of AP Side Effects (Among Participants Who Experienced Side Effect). AP = Antipsychotic. Note: The sample size for each side effect only includes participants who reported experiencing the side effect. Side effects are listed from left to right in descending order of percentage of participants who reported “very bothersome” or “extremely bothersome.” Data are based on 198 participants who completed this item; two participants’ responses were not recorded due to technology problems, and have been excluded from frequency calculations

Almost all (98%) participants experienced at least one side effect of current or previous AP treatment (Fig. 1). Each of the 12 listed side effects was experienced by nearly two-thirds or more of the sample, with the least frequently endorsed side effect reported by 62% of participants. The five most commonly experienced side effects were feeling drowsy or tired (83%), feeling a lack of emotion (79%), anxiety (79%), dry mouth (76%), and weight gain (76%).

Side effects rated as “very bothersome” or “extremely bothersome” by more than half of participants were weight gain (68%), trouble concentrating (53%), and feeling drowsy or tired (52%). The side effect with the highest proportion of being rated “extremely bothersome” was weight gain (44%); the next highest were trouble concentrating, restlessness, and sexual dysfunction (23–24%).

#### Perceived impacts on social functioning

AP side effects commonly impaired interactions with others, including family and romantic partners, and caused participants to feel embarrassed in front of other people (Table 2).

**Table 2:** Perceived Impacts of AP Side Effects on Social Functioning (*N* = 195)

AP Side Effect	N^**a**^	Impacted Interactions with People (not including family)	Impacted Relationships with Family	Impacted Romantic Relationships	Made You Feel Embarrassed in Front of Other People
Feeling drowsy or tired	165	**75 (45.5%)**	80 (48.5%)	64 (38.8%)	33 (20.0%)
Feeling a lack of emotion	157	69 (43.9%)	**93 (59.2%)**	**75 (47.8%)**	32 (20.4%)
Anxiety	156	**104 (66.7%)**	**95 (60.9%)**	**77 (49.4%)**	**76 (48.7%)**
Weight gain	150	42 (28.0%)	45 (30.0%)	59 (39.3%)	**73 (48.7%)**
Dry mouth	150	30 (20.0%)	31 (20.7%)	19 (12.7%)	31 (20.7%)
Restlessness	146	61 (41.8%)	48 (32.9%)	44 (30.1%)	36 (24.7%)
Trouble concentrating	141	**72 (51.1%)**	69 (48.9%)	52 (36.9%)	47 (33.3%)
Feeling like a “zombie”	137	58 (42.3%)	**69 (50.4%)**	48 (35.0%)	35 (25.5%)
Digestive (gastrointestinal) problems, including nausea	135	31 (23.0%)	34 (25.2%)	27 (20.0%)	40 (29.6%)
Sexual dysfunction (for example, loss of sex drive or performance issues)	133	12 (9.0%)	13 (9.8%)	**84 (63.2%)**	11 (8.3%)
Involuntary spasms, movements, twitching, or stiffness	123	34 (27.6%)	35 (28.5%)	24 (19.5%)	**44 (35.8%)**
Dizziness/fainting	123	27 (22.0%)	23 (18.7%)	21 (17.1%)	28 (22.8%)

*AP* Antipsychotic

^a^ N only includes participants who experienced the listed AP medication side effect

Percentages are calculated based on the N for each side effect

Side effects are listed in order of descending overall proportion of participants who reported experiencing the side effect

The three most frequently selected side effects for each of the four categories are bolded

Anxiety had the greatest perceived impact on social functioning: most participants who experienced anxiety reported that it impacted interactions with both family and non-family members (61 and 67%, respectively). For non-family interactions, trouble concentrating, feeling drowsy or tired, lack of emotion, “feeling like a ‘zombie,’” and restlessness followed anxiety as the most commonly impactful side effects. Relationships with family were also impaired by lack of emotion, “feeling like a ‘zombie,’” trouble concentrating, and feeling drowsy or tired. Romantic interactions were most commonly impacted by sexual dysfunction, anxiety, lack of emotion, weight gain, and feeling drowsy or tired. The side effects that most frequently led to embarrassment were anxiety and weight gain, followed by involuntary spasms or movements and trouble concentrating. Across these social outcomes, anxiety and lack of emotion were the AP side effects that were most consistently perceived as impacting social functioning.

#### Impact of AP side effects on medication regimen

Stopping APs was reported by nearly two-thirds (66%) of participants, more often with MHCP agreement than without (51% vs. 44%). About half (52%) reported reducing (i.e., taking less of or taking less often) the prescribed AP.

Regardless of MHCP agreement, the most common reason for stopping APs was dislike of side effects (48%), while 24% reported stopping because the medication did not help with symptoms.

For those who reduced their AP dosage, side effects were the most common cited reason with MHCP agreement (46%), and the second most cited for those who did so without MHCP agreement (48%). Among this latter group, the most common reason for taking APs less often was forgetting to take their medication (53%). As with stopping, about one-quarter (24%) of participants reported reducing APs because the medication did not help with symptoms.

##### Side effects associated with changes to medication regimen

Among participants reporting stopping or reducing medication due to side effects (with or without agreement with their MHCP), the side effects most frequently reported as the reason for changes in AP regimens were “feeling like a ‘zombie’” (29%), feeling drowsy or tired (25%), weight gain (24%), and a lack of emotion (21%).

**Fig. 2:**
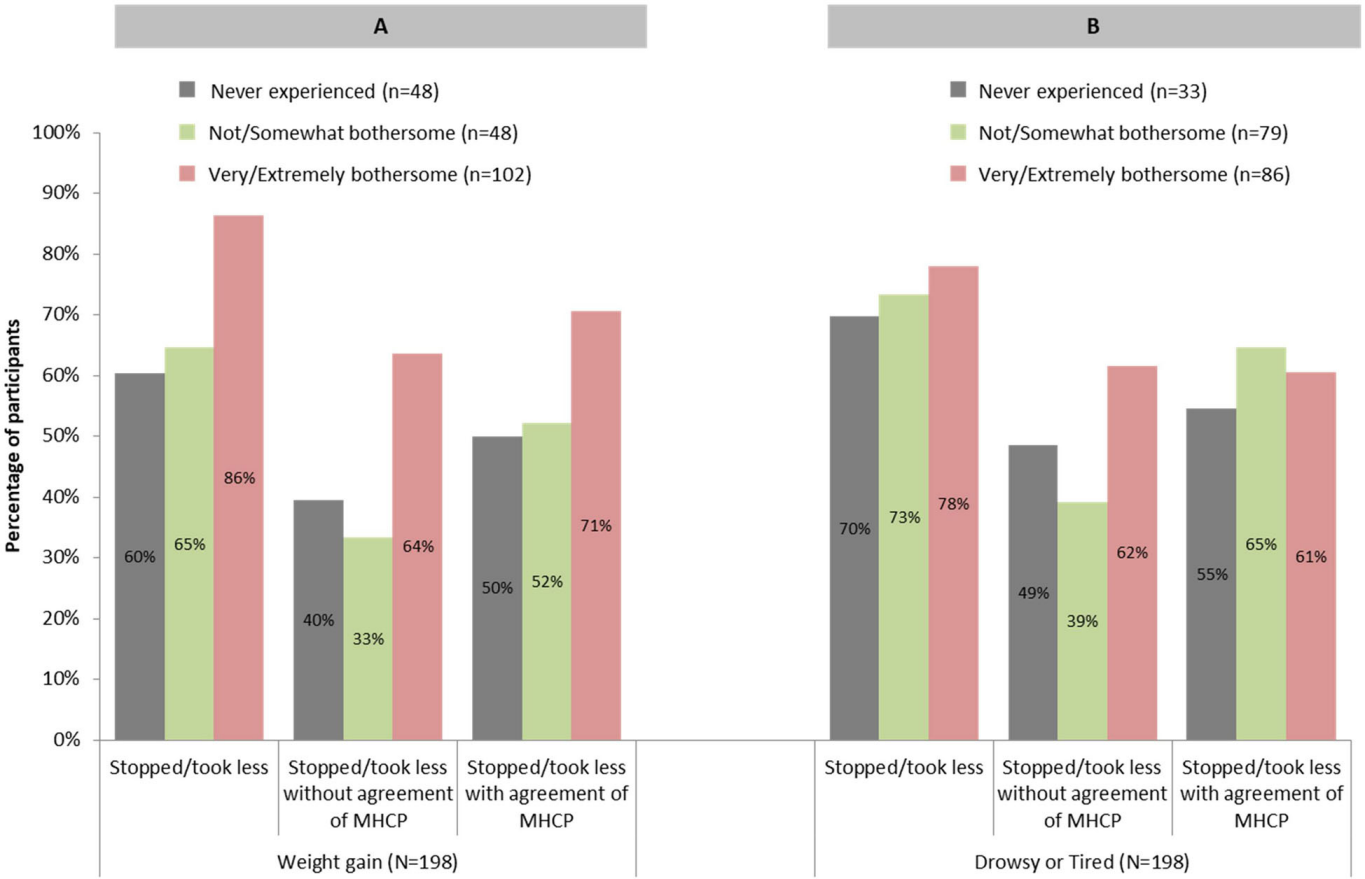
Changes to Medication Regimen by Three Categories of Side Effect Experience (*N* = 198). MHCP = Mental Health Care Professional

A larger percentage of participants had experienced weight gain and found it to be “very/extremely bothersome” stopped or reduced their AP, with or without MHCP agreement, than did participants who rated their weight gain as “not/somewhat bothersome” or who had not experienced weight gain (Fig. 2a).

In contrast, participants who either stopped or reduced APs did not as greatly differ as a function of their prior experiences of the side effect feeling drowsy or tired (Fig. 2b). A larger proportion of participants who found feeling drowsy or tired to be “very/extremely bothersome” stopped APs without agreement of their MHCP as compared to participants who either found this side effect to be “not/somewhat bothersome” or had never experienced this side effect. However, the proportion of participants who stopped APs with agreement of their MHCP did not greatly differ based on participants’ past experiences with, or extent of bothersomeness of, feeling drowsy or tired.

#### Job performance and relationships with co-workers

Participants reported BD-I and its treatment had altered or limited their employment: nearly half of participants (44%) had left a job due to BD-I or its treatment, 33% had taken leaves of absence, 30% had been fired from a job, and 28% had reduced or changed their work hours.

BD-I symptoms were perceived as having negatively impacted relationships with co-workers and job performance (63 and 69%, respectively). AP side effects were also reported to have impacted these aspects of employment, but to a lesser degree (37 and 49%, respectively).

**Fig. 3:**
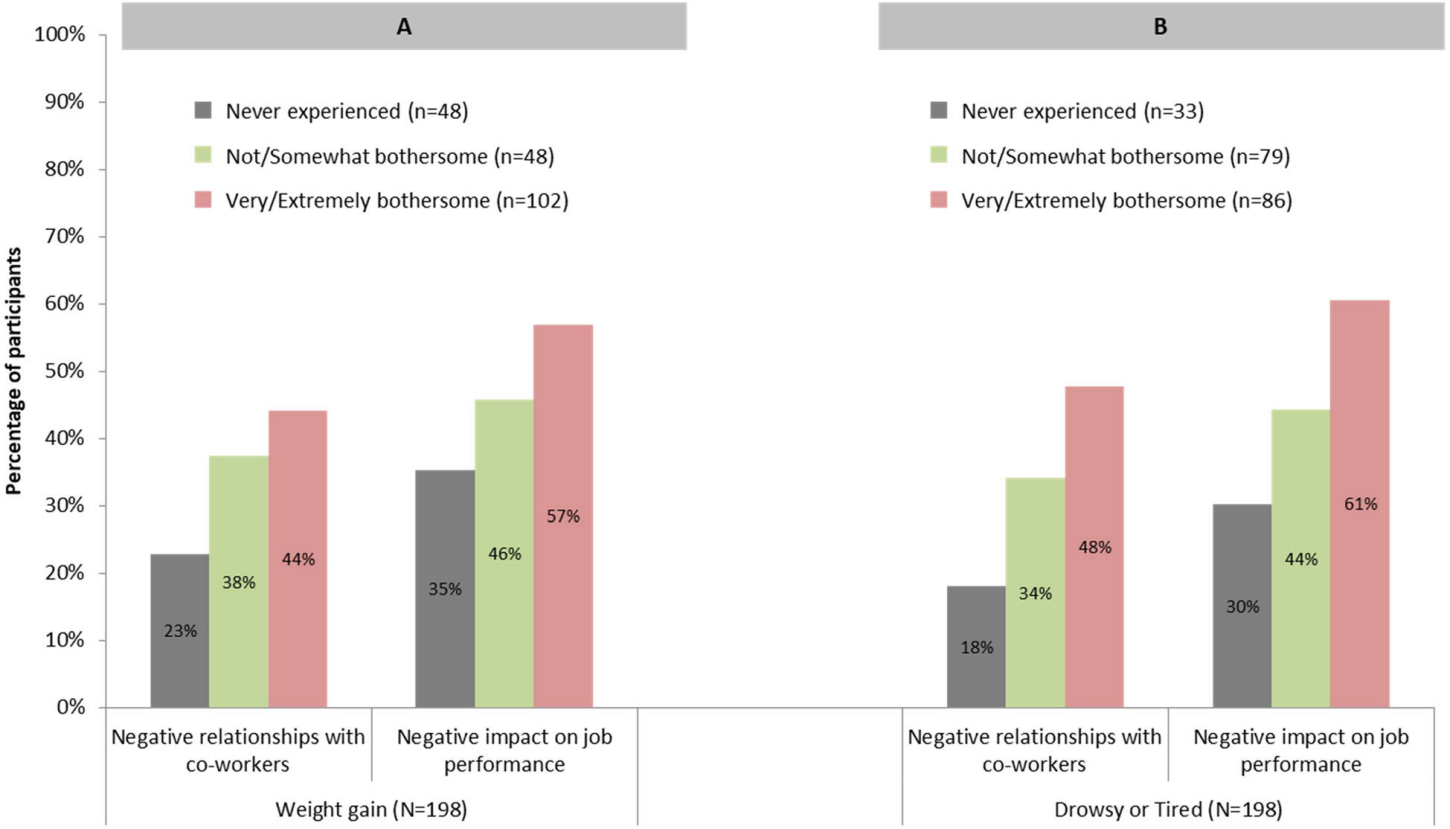
Perceived Impact of AP Medication Side Effects on Employment by Three Categories of Side Effect Experience (*N* = 198). AP = Antipsychotic

Participants who experienced “very/extremely bothersome” weight gain reported more negative impacts on relationships with co-workers and job performance compared to those for whom weight gain was not as bothersome or those who had not experienced weight gain (Fig. 3a). Similar patterns were observed for feeling drowsy or tired (Fig. 3b).

### Preferences for a hypothetical new AP

When participants were asked which manic symptoms they would most want controlled by a hypothetical new AP (choosing up to five from the 16 listed, or “other”), the most frequently chosen were anxiety (69%), feeling angry or irritated with friends, family, or co-workers (52%), feeling restless (43%), racing thoughts (42%), and difficulty paying attention (42%).

Side effects participants would most like to avoid in a hypothetical new AP were anxiety (50%), weight gain (48%), and “feeling like a ‘zombie’” (47%).

Sixty-six percent of participants who experienced “very/extremely bothersome” weight gain chose weight gain as one of their top five side effects to most avoid, compared to 42% who were less bothered by weight gain, and 18% who had not experienced this side effect.

Forty-one percent of participants who experienced “very/extremely bothersome” drowsiness or tiredness chose feeling drowsy or tired as one of their top five side effects that they wanted to avoid, as compared to 18% who were less bothered by this side effect, and 18% who had not experienced this side effect.

#### Trade-offs between symptom management and side effects

**Fig. 4:**
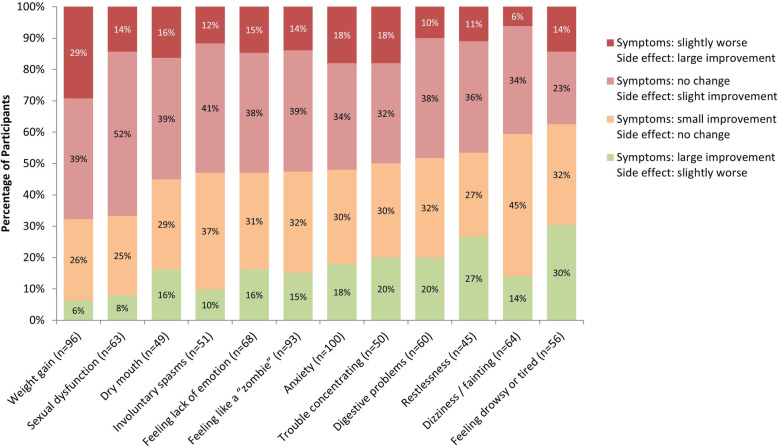
Trade-offs between Symptom Relief and Side Effects in an AP Medication. AP = Antipsychotic. N for each side effect was based on the number of participants who chose that side effect as one they most wanted to avoid in a hypothetical new AP. Note: Side effects are listed from left to right in descending order of percentage of participants who reported “no change in symptoms but small improvement in <side effect>” or “slightly worse symptoms but a large improvement in <side effect>”

When considering trade-offs between symptom management and side effects of a hypothetical new AP, as compared to their experiences with current or most recent AP medication, participants most often endorsed small improvements in symptoms or side effects with no change in the other, as opposed to large improvements in symptoms or side effects at the expense of slight worsening in the other (Fig. 4). Fewer participants endorsed large improvements in one at the expense of slight worsening in the other.

In contrast, for weight gain and sexual dysfunction, side effect improvement was endorsed more frequently than improvement in symptom management compared to their current or most recent AP medication. In fact, almost a third of participants selected a hypothetical AP with slightly worse symptom management if it was accompanied by a large improvement in weight gain (i.e., reduced propensity for weight gain). On the other hand, considering restlessness, dizziness/fainting, and feeling drowsy or tired, larger proportions of participants favored symptom improvement over improvements in side effects.

## Discussion

This cross-sectional observational survey captured the experiences of 200 adults with BD-I with a history of recent AP use within the last 12 months, with more than 80% who were on AP treatment at the time of the survey. Half of participants reported a manic episode and two-thirds of participants reported a depressive episode within the past month. The most frequently reported symptoms were anxiety, decreased pleasure or interest in activities, and decreased sleep.

Overall, participants appeared moderately satisfied with their current medication, though somewhat dissatisfied with the side effects. Almost all participants reported experiencing side effects of APs. Feeling drowsy or tired was the most frequently reported, followed by lack of emotion, anxiety, dry mouth, and weight gain.

Previous research has documented negative impacts of BD on relationships with family and friends, and desire to engage in social activities [19, 20]. The results of this survey suggest that side effects of APs contribute to impairment in social functioning including how individuals with BD interact with family and romantic partners, and how side effects contribute to stigma and embarrassment.

In this survey sample, many participants reported stopping or reducing APs (with or without agreement of their MHCP). “Not liking” AP side effects was the most commonly reported reason for stopping and/or reducing medication. Among those who stopped or reduced APs, “feeling like a ‘zombie,’” feeling drowsy or tired, weight gain, and lack of emotion were most frequently cited. Other studies document non-adherence rates for APs and other psychiatric medications in individuals with BD, ranging from 20 to 70% [10, 13, 21–24]. Consistent with our findings, others report side effects contribute to non-adherence [25, 26].

BD-I symptoms had substantial negative impact on work experiences, including limiting employment (e.g., changing jobs, reducing working hours, disability leave) and impairing job performance and relationships with co-workers. These findings suggest that in addition to the disease itself, AP side effects also negatively impact job performance and relationships with co-workers for more than one-third of participants.

When considering hypothetical new AP treatment, participants most favored avoiding anxiety, weight gain, and “feeling like a ‘zombie.’” Considering trade-offs between symptom management and side effects in comparison to their experiences with current or most recent AP medication, participants often chose improvements in symptoms at the cost of worsening or no change in side effects. However, for some side effects, notably weight gain and sexual dysfunction, a relatively large proportion of participants favored improvements in the side effect over improvements in symptoms. In fact, nearly one-third of participants seeking improvements in weight gain would accept slight worsening in disease symptoms experienced while taking their current or most recent medication. That is, some participants were willing to accept a new AP that had a better side effect profile for weight gain at the expense of its efficacy.

Further, weight gain was rated as the most bothersome side effect. In addition, participants who had previously experienced highly bothersome weight gain more often stopped or reduced their AP due to not liking side effects than participants who had found it less bothersome. These participants also more frequently reported negative impacts of side effects on job performance and relationships with co-workers. Many APs are associated with high incidence rates of metabolic problems and weight gain [27, 28]. Significant weight gain has been associated with exacerbated symptoms and hinders quality of life among individuals with BD-I [29]. Further, weight gain has been reported to be a predictor of whether individuals would be adherent to a hypothetical BD medication [8]. Thus, there is a strong need for highly efficacious APs that minimize the side effect of weight gain.

A discrepancy in frequency of side effects was observed between the current survey and the qualitative study that preceded it [18]. Specifically, a notably larger proportion of participants cited sexual dysfunction as an AP side effect compared to focus group participants (68% vs. 46%). This proportion was also greater than sexual dysfunction/dissatisfaction observed in prior research using paper-pencil questionnaires in female BD patients, of whom many had history of AP use [30]. This difference may be a function of the anonymity of survey responses using the observational, online questionnaire methodology. Using this method removes the influence of social stigma for reporting of what may be considered a sensitive topic, particularly when compared to other approaches for data collection (e.g., interviews/focus groups; communicating with MHCP in a clinical setting). The potential for different patterns of responses between these approaches underscores the need to use multiple techniques, and the importance of using techniques that afford anonymity of responses, to adequately capture patient experiences in research settings. However, MHCPs should expect that in clinical practice, where anonymity is not possible, stigmatized symptoms and side effects such as sexual dysfunction may be underreported by patients.

The frequency of participants who reported experiencing a depressive episode (67%) or a manic episode (51%) in the previous month was higher than would be expected. Prior research indicates that patients with BD-I experience an average of two episodes per year [31], a lower rate than what is reported in this study. One potential explanation for the likely over-reporting of depressive or manic episodes is that participants may have misinterpreted the items as asking about recent depressive or manic *symptoms* rather than episodes, despite the use of the word “episode” in the items. It is also possible that participants may have interpreted an episode more broadly than was intended, which was as a clinically defined depressive or manic episode [1]. For example, a participant who experienced low mood during part of 1 day might have considered that as an “episode.” These results point to the potential risk of using clinical terminology, without definition, when collecting patient-reported data, as it could produce biased results due to misinterpretation.

Other limitations of this survey should be considered. These data are descriptive and cannot be used to assess causality. Although sampling quotas for age and gender were implemented, this sample may not be representative of the larger population of people with BD-I. Educational attainment in this sample was relatively high considering previous findings that individuals with BD have lower educational attainment when compared to the general population [32]. However, the sample had similar age, gender, race, and educational attainment distributions as reported in other large observational studies of BD [33–35], indicating that any differences in educational attainment or other demographic characteristics may be a function of the survey methodology. That is, the recruitment of participants using online panels may have biased the types of individuals with BD-I who participated in the survey.

Further, we excluded patients who had been recently hospitalized for their BD-I to minimize the possibility of including participants who might currently be experiencing psychosis, and likely would not be able to provide accurate responses to the survey. In addition, prior research indicates that patients with severe acute symptoms often are either not taking AP medication, or may be experiencing adjustments in medication regimen [36]. Thus, this exclusion criterion was also applied so that participants’ responses would focus on their maintenance drug treatment experiences rather than experiences based mostly upon acute underlying illness or the immediate effects of drug titration. Our exclusion of these patients, however, limits our ability to generalize these findings to the full spectrum of individuals with BD-I who may be in various stages of treatment and recovery.

Participants’ diagnosis of BD-I by a physician was self-reported and was not externally verified. As with all surveys, recall bias may have impacted responses.

Given that combination treatment with multiple psychotropic medications is common in BD-I [37, 38], and use of non-AP medications (e.g., mood stabilizers, antidepressants) was not captured in the survey, it remains unclear whether the side effects reported by participants are specifically attributable to the reported AP, or to any other types of medications they may have been taking concurrently. Because we did not collect data regarding other psychiatric medications, we were not able to differentiate between side effects that may be due to these other medications from those due to AP treatment. Moreover, experience of symptoms and side effects can overlap in presentation (e.g., anxiety was reported as both a symptom and a side effect), making it difficult for participants to disentangle some of their experiences when responding to survey items. As a result of these issues, it is possible that participants over-reported their experiences of side effects due to their AP medication in the survey.

Despite these limitations, the study has several strengths. The survey content was informed by focus groups of participants with BD-I [18] and input from expert clinicians, psychometricians, and representatives from national mental health patient advocacy groups. This type of patient-centered evidence that is consistent with the FDA patient-focused drug development approach, for which the primary goal is to better incorporate the patient’s voice in drug development and evaluation.

## Conclusions

Side effects of APs are a considerable concern for people living with BD-I, and have a perceived impact on social functioning, medication adherence, and work. These findings highlight patients’ perspectives on the unmet need for new APs for BD-I that have more favorable risk-benefit profiles.

## Supplementary information

**Additional file 1.** Original survey items.

**Additional file 2.** Supplementary tables and figures.

## Data Availability

The data collected in this study are proprietary to Alkermes, Inc. Alkermes, Inc. is committed to public sharing of data in accordance with applicable regulations and laws.

## Abbreviations

AP: Antipsychotic
BD: Bipolar disorder
BD-I: Bipolar I disorder
EPS: Extrapyramidal symptoms
FGA: First-generation antipsychotics
ICF: Informed consent form
MHCP: Mental health care professional
SAP: Statistical analysis plan
SD: Standard deviation
SGA: Second-generation antipsychotics
TSQMvII: Treatment Satisfaction Questionnaire for Medication Version II
US: United States

## Footnotes

1 MHCP refers to any mental health care professional, not just the MHCP who prescribed a patient’s AP treatment regimen.

2 For the qualitative study, three 90-min focus groups (*N* = 24; 8 participants per group) were conducted with participants with BD-I meeting similar eligibility criteria as the current survey [18].
