# Supplementary material for: Antipsychotic treatment experiences of people with bipolar I disorder: patient perspectives from an online survey
Source: BMC Psychiatry. 2020 Jul 10;20:354. doi: 10.1186/s12888-020-02767-x (PMC7371473; doi:10.1186/s12888-020-02767-x)
Supplement: Supplementary file 1 — Additional file 1. Original survey items. [file 12888_2020_2767_MOESM1_ESM.docx]

**Original Survey Items**

**Screener**

1. What is your date of birth?*

MM/DD/YYYY

1. Do you currently live in the United States?*

Yes 🞏

No 🞏

1. What is your gender?

Male 🞏

Female 🞏

Other 🞏

1. Have you ever been diagnosed by a healthcare provider (such as your primary care doctor, a psychiatrist, or a psychologist) with any of the following conditions (select all that apply)?*

Bipolar I disorder 🞏

Bipolar II disorder 🞏

Cyclothymic disorder 🞏

Unspecified bipolar disorder 🞏

None of the above 🞏

1. How long ago were you diagnosed with bipolar I disorder?*

Less than one year ago 🞏

More than 1 but less than 2 years ago 🞏

More than 2 but less than 3 years ago 🞏

More than 3 but less than 5 years ago 🞏

More than 5 but less than 10 years ago 🞏

More than 10 years ago 🞏

1. Have you ever been diagnosed by a healthcare provider (such as your primary care doctor, a psychiatrist, or a psychologist) with schizophrenia or schizoaffective disorder?*

Yes 🞏

No 🞏

1. Have you received treatment from a mental health professional (such as a psychiatrist, psychologist, social worker, or psychiatric nurse) for bipolar disorder at any time during the past three months?*

Yes 🞏

No 🞏

1. Oral antipsychotic medications are medications that you take by mouth, such as swallowing a pill or liquid, and include the following:

| aripiprazole (Abilify) |  | molindone (Moban) |
| --- | --- | --- |
| asenapine (Saphris) |  | olanzapine (Zyprexa) |
| brexpiprazole (Rexulti) |  | paliperidone (Invega) |
| cariprazine (Vraylar) |  | perphenazine (Trilafon) |
| chlorpromazine (Thorazine) |  | pimozide (Orap) |
| clozapine (Clozaril, Fazaclo, Versacloz) |  | quetiapine (Seroquel) |
| fluoxetine & olanzapine (Symbax) |  | risperidone (Risperdal) |
| fluphenazine (Permitil, Prolixin) |  | thioridazine (Mellaril, Melleril) |
| haloperidol (Haldol) |  | thiothixine (Navane) |
| iloperidone (Fanapt) |  | trifluoperazine (Stelazine, Vesprin) |
| loxapine (Loxitane) |  | ziprasidone (Geodon) |
| lurasidone (Latuda) |  |  |

Have you taken an oral antipsychotic medication for clinically diagnosed bipolar disorder for at least one week within the past year?

Yes 🞏

No 🞏

1. Have you been hospitalized because of psychiatric problems at any time during the past three months?*

Yes 🞏

No 🞏

1. Have you participated in a clinical trial for bipolar disorder at any time during the past year?*

Yes 🞏

No 🞏

* Responses to these questions were used to determine eligibility to participate in this study.

**Symptoms**

1. When was the last time you experienced a manic episode, such as a lasting period of racing thoughts, extreme energy, or ‘wired’ feelings?

Within the past month 🞏

1 to less than 2 months ago 🞏

2 to less than 3 months ago 🞏

3 to less than 6 months ago 🞏

6 to less than 12 months ago 🞏

More than 1 year ago 🞏

1. When was the last time you experienced a depressive episode, such as a lasting period of low mood or loss of pleasure or interest in activities?

Within the past month 🞏

1 to less than 2 months ago 🞏

2 to less than 3 months ago 🞏

3 to less than 6 months ago 🞏

6 to less than 12 months ago 🞏

More than 1 year ago 🞏

1. Please think about the last time you had a manic episode. Please select all of the symptoms you experienced from the list below.

Anxiety 🞏

Decreased sleep 🞏

Difficulty paying attention to one thing 🞏

Drug/alcohol use 🞏

Extreme focus on a specific task, even to the point of ignoring other important responsibilities 🞏

Extremely high sex drive or engaging in sexual behaviors that are unusual for you 🞏

Feeling angry or irritated with friends, family, or co-workers 🞏

Feeling more important or powerful with respect to others than you have in the past 🞏

Feeling that others are out to get you 🞏

Feeling restless 🞏

High mood, such as extreme joy or excitement 🞏

Increased or faster talking 🞏

Racing thoughts 🞏

Risky or impulsive behaviors, such as spending money you don’t have or making major life changes 🞏

without much thought 🞏

Thoughts of your own death or a suicide attempt 🞏

Visual hallucinations 🞏

Other ____________ 🞏

1. Please think about the last time you had a depressive episode. Please select all of the symptoms you experienced most of the day or nearly every day from the list below.

Anxiety 🞏

Avoiding interactions with friends, family, or co-workers 🞏

Changes in weight or appetite (increased or decreased) 🞏

Decreased pleasure or interest in activities 🞏

Decreased sleep or sleeping too much 🞏

Difficulty thinking, concentrating, or making decisions 🞏

Feeling that life is meaningless 🞏

Feeling tired or loss of energy 🞏

Feeling worthless or guilty 🞏

Loss of sex drive 🞏

Low mood, such as feeling sad or empty 🞏

Slowing down of thoughts or movement 🞏

Thoughts of your own death or a suicide attempt 🞏

Other ___________ 🞏

**Treatment History and Side Effects**

Please tell us about your use of **oral** antipsychotic medication using the grid below. An oral antipsychotic medication is an antipsychotic medication that you take by mouth, such as swallowing a pill or liquid. For each medication, pick the response that describes your use.

|  | Never taken | Currently taking | Not currently taking, but have taken within the last year | Not currently taking, but took more than one year ago |
| --- | --- | --- | --- | --- |
| 1. Aripiprazole (Abilify) | 🞏 | 🞏 | 🞏 | 🞏 |
| 1. Asenapine (Saphris) | 🞏 | 🞏 | 🞏 | 🞏 |
| 1. Brexpiprazole (Rexulti) | 🞏 | 🞏 | 🞏 | 🞏 |
| 1. Cariprazine (Vraylar) | 🞏 | 🞏 | 🞏 | 🞏 |
| 1. Chlorpromazine (Thorazine) | 🞏 | 🞏 | 🞏 | 🞏 |
| 1. Clozapine (Clozaril, Fazaclo, Versacloz) | 🞏 | 🞏 | 🞏 | 🞏 |
| 1. Fluoxetine & Olanzapine (Symbax) | 🞏 | 🞏 | 🞏 | 🞏 |
| 1. Fluphenazine (Permitil, Prolixin) | 🞏 | 🞏 | 🞏 | 🞏 |
| 1. Haloperidol (Haldol) | 🞏 | 🞏 | 🞏 | 🞏 |
| 1. Iloperidone (Fanapt) | 🞏 | 🞏 | 🞏 | 🞏 |
| 1. Loxapine (Loxitane) | 🞏 | 🞏 | 🞏 | 🞏 |
| 1. Lurasidone (Latuda) | 🞏 | 🞏 | 🞏 | 🞏 |
| 1. Molindone (Moban) | 🞏 | 🞏 | 🞏 | 🞏 |
| 1. Olanzapine (Zyprexa) | 🞏 | 🞏 | 🞏 | 🞏 |
| 1. Paliperidone (Invega) | 🞏 | 🞏 | 🞏 | 🞏 |
| 1. Perphenazine (Trilafon) | 🞏 | 🞏 | 🞏 | 🞏 |
| 1. Pimozide (Orap) | 🞏 | 🞏 | 🞏 | 🞏 |
| 1. Quetiapine (Seroquel) | 🞏 | 🞏 | 🞏 | 🞏 |
| 1. Risperidone (Risperdal) | 🞏 | 🞏 | 🞏 | 🞏 |
| 1. Thioridazine (Mellaril, Melleril) | 🞏 | 🞏 | 🞏 | 🞏 |
| 1. Thiothixine (Navane) | 🞏 | 🞏 | 🞏 | 🞏 |
| 1. Trifluoperazine (Stelazine, Vesprin) | 🞏 | 🞏 | 🞏 | 🞏 |
| 1. Ziprasidone (Geodon) | 🞏 | 🞏 | 🞏 | 🞏 |

*If no medications are selected for “currently taking”, skip TSQMvII, otherwise, show TSQMvII.*

*On each subsequent page, please include hover text over every instance of “oral antipsychotic medication” that states “To see the list of oral antipsychotic medications, click the question mark at the top of the page.” Clicking on the question mark will open a pop up window with a table of the oral antipsychotic medications listed above.*

**We would like to know about both the positive and negative experiences associated with your oral antipsychotic medication. When responding, please think about all of the oral antipsychotic medications that you have been on rather than any specific medication.**

When you think about all the oral antipsychotic medications you have taken for bipolar disorder, how bothersome have you found the following side effects?

*If participant chooses “I have not experienced this side effect” for any of the side effects mentioned in items 28-39 below, eliminate the corresponding side effect response options from items 41-52, 62*

|  | I have not experienced this side effect | Not bothersome | Somewhat bothersome | Very bothersome | Extremely bothersome |
| --- | --- | --- | --- | --- | --- |
| 1. Anxiety | 🞏 | 🞏 | 🞏 | 🞏 | 🞏 |
| 1. Digestive (gastrointestinal) problems, including nausea | 🞏 | 🞏 | 🞏 | 🞏 | 🞏 |
| 1. Dizziness / fainting | 🞏 | 🞏 | 🞏 | 🞏 | 🞏 |
| 1. Dry mouth | 🞏 | 🞏 | 🞏 | 🞏 | 🞏 |
| 1. Feeling a lack of emotion | 🞏 | 🞏 | 🞏 | 🞏 | 🞏 |
| 1. Feeling drowsy or tired | 🞏 | 🞏 | 🞏 | 🞏 | 🞏 |
| 1. Feeling like a “zombie” | 🞏 | 🞏 | 🞏 | 🞏 | 🞏 |
| 1. Involuntary spasms, movements, twitching, or stiffness | 🞏 | 🞏 | 🞏 | 🞏 | 🞏 |
| 1. Restlessness | 🞏 | 🞏 | 🞏 | 🞏 | 🞏 |
| 1. Sexual dysfunction (for example, loss of sex drive or performance issues) | 🞏 | 🞏 | 🞏 | 🞏 | 🞏 |
| 1. Trouble concentrating | 🞏 | 🞏 | 🞏 | 🞏 | 🞏 |
| 1. Weight gain | 🞏 | 🞏 | 🞏 | 🞏 | 🞏 |

Have you had any other side effects from your oral antipsychotic medications that we not ask about? If so, please type the side effect in the space below, and rate how bothersome this side effect has been for you. You can add more than one side effect if needed.

1. Other _____________________

Not bothersome 🞏

Somewhat bothersome 🞏

Very bothersome 🞏

Extremely bothersome 🞏

*Include option to add another side effect.*

*If participants include an “other” side effect in item 40, display this with list of side effects in items 53, 62 & 65.*

Which oral antipsychotic medication side effects…(Select all that apply.)

|  | Have impacted your interactions with other people (not including family) | Have impacted your relationships with family | Have impacted your romantic relationships | Make you feel embarrassed in front of other people |
| --- | --- | --- | --- | --- |
| 1. Anxiety | 🞏 | 🞏 | 🞏 | 🞏 |
| 1. Digestive (gastrointestinal) problems, including nausea | 🞏 | 🞏 | 🞏 | 🞏 |
| 1. Dizziness / fainting | 🞏 | 🞏 | 🞏 | 🞏 |
| 1. Dry mouth | 🞏 | 🞏 | 🞏 | 🞏 |
| 1. Feeling a lack of emotion | 🞏 | 🞏 | 🞏 | 🞏 |
| 1. Feeling drowsy or tired | 🞏 | 🞏 | 🞏 | 🞏 |
| 1. Feeling like a “zombie” | 🞏 | 🞏 | 🞏 | 🞏 |
| 1. Involuntary spasms, movements, twitching, or stiffness | 🞏 | 🞏 | 🞏 | 🞏 |
| 1. Restlessness | 🞏 | 🞏 | 🞏 | 🞏 |
| 1. Sexual dysfunction (for example, loss of sex drive or performance issues) | 🞏 | 🞏 | 🞏 | 🞏 |
| 1. Trouble concentrating | 🞏 | 🞏 | 🞏 | 🞏 |
| 1. Weight gain | 🞏 | 🞏 | 🞏 | 🞏 |
| 1. Other ____________ | 🞏 | 🞏 | 🞏 | 🞏 |

**Interactions with the healthcare system**

**The next questions are about your experiences with mental health care professionals (e.g., psychiatrist, psychologist, primary health care provider, peer specialist, social worker).**

1. How satisfied are you with your conversations with mental health care professionals about oral antipsychotic medications for bipolar disorder?

Completely satisfied 🞏

Generally satisfied 🞏

Undecided (neither satisfied nor unsatisfied) 🞏

Generally unsatisfied 🞏

Completely unsatisfied 🞏

1. How often do you feel that you and your mental health care professionals work together as a team in coming up with your oral antipsychotic medication plan?

Always 🞏

Very often 🞏

Often 🞏

Sometimes 🞏

Never 🞏

**Treatment Adherence**

1. Have you ever stopped taking your oral antipsychotic medication **with the agreement** of your mental health care professional(s)?

Yes 🞏

No 🞏

1. Have you ever stopped taking your oral antipsychotic medication even if your mental health care professional(s) **did not recommend it**?

Yes 🞏

No 🞏

*If responses to items 56 and 57 are both “no,” skip to item 59.*

1. What are some reasons why you stopped taking your oral antipsychotic medication? (Select all that apply.)

Changed to a new medication 🞏

I did not like taking medication 🞏

I did not like the side effects of the medication 🞏

I did not think that I needed medication 🞏

I did not want the medication to control my symptoms 🞏

I felt ashamed or embarrassed about taking medication 🞏

I needed to take the medication too often 🞏

My symptoms improved, so I did not need the medication anymore 🞏

The medication did not help my symptoms 🞏

The cost of the medication was too high 🞏

Other ____________________________ 🞏

1. Have you ever taken less of your oral antipsychotic medication or taken it less often **with the agreement** of your mental health care professional(s)?

Yes 🞏

No 🞏

1. Have you ever taken less of your oral antipsychotic medication or taken it less often even if your mental health care professional(s) **did not recommend it**?

Yes 🞏

No 🞏

*If responses to items 59 and 60 are both “no,” skip to item 63.*

1. What are some reasons you have taken less of your oral antipsychotic medication or have taken it less often? (Select all that apply.)

I did not like taking medication 🞏

I did not like the side effects of the medication 🞏

I did not think that I needed medication 🞏

I did not want the medication to control my symptoms 🞏

I felt ashamed or embarrassed about taking medication 🞏

I forgot to take my medication 🞏

I needed to take the medication too often 🞏

My symptoms improved, so I did not need as much medication anymore 🞏

The medication did not help my symptoms 🞏

The cost of the medication was too high 🞏

Other ____________________________ 🞏

*If the response “I did not like the side effects of the medication” was chosen for item 58 and/or 61, please proceed to item 62. Otherwise proceed to item 63.*

1. Please select the side effects that made you want to stop or take less of your oral antipsychotic medication. (Select all that apply.)

Anxiety 🞏

Digestive (gastrointestinal) problems, including nausea 🞏

Dizziness / fainting 🞏

Dry mouth 🞏

Feeling a lack of emotion 🞏

Feeling drowsy or tired 🞏

Feeling like a “zombie” 🞏

Involuntary spasms, movements, twitching, or stiffness 🞏

Restlessness 🞏

Sexual dysfunction (for example, loss of sex drive or performance issues) 🞏

Trouble concentrating 🞏

Weight gain 🞏

Other _________________ 🞏

None 🞏

**Treatment Expectations**

**The next few questions are about what you would like to see in a new oral antipsychotic medication for bipolar disorder.**

1. In considering a new oral antipsychotic medication for treatment of bipolar disorder, which symptoms would you most want to see controlled? (Select up to five responses.)

Anxiety 🞏

Decreased sleep 🞏

Difficulty paying attention to one thing 🞏

Extreme focus on a specific task, even to the point of ignoring important responsibilities 🞏

Extremely high sex drive or engaging in sexual behaviors that are unusual for you 🞏

Feeling angry or irritated with friends, family, or co-workers 🞏

Feeling more important or powerful with respect to others than you have in the past 🞏

Feeling that others are out to get you 🞏

Feeling restless 🞏

Feeling that others are out to get you 🞏

High mood, such as extreme joy or excitement 🞏

Increased or faster talking 🞏

Racing thoughts 🞏

Risky or impulsive behaviors, such as spending money you don’t have or making major life changes

without much thought 🞏

Visual hallucinations 🞏

Other_________________ 🞏

None of the above 🞏

1. In considering a new oral antipsychotic medication for bipolar disorder, which side effects would you most want to **avoid**? (Select up to five responses.)

Anxiety 🞏

Digestive (gastrointestinal) problems, including nausea 🞏

Dizziness / fainting 🞏

Dry mouth 🞏

Feeling a lack of emotion 🞏

Feeling drowsy or tired 🞏

Feeling like a “zombie” 🞏

Involuntary spasms, movements, twitching, or stiffness 🞏

Restlessness 🞏

Sexual dysfunction (for example, loss of sex drive or performance issues) 🞏

Trouble concentrating 🞏

Weight gain 🞏

Other _________________ 🞏

None 🞏

*Among items 65 through 77, only present those for side effects that match responses chosen in item 64. Thus, no more than five of items 65 through 77 should be presented.*

**In the next questions, you will be presented with imaginary "scenarios" about a new oral antipsychotic medication.**

**Each scenario describes the new medication’s effect on bipolar disorder symptoms and also the severity of the medication side effects that you chose in the previous question.**

**For each scenario, please choose the option that you most prefer.**

1. Compared to your current or most recent oral antipsychotic medication, which of the following would you prefer?

**Large improvement** in symptoms, but **slightly worse** anxiety 🞏

**Small improvement** in symptoms, but **no change** in anxiety 🞏

**No change** in symptoms, but **small improvement** in anxiety 🞏

**Slightly worse** symptoms, but **large improvement** in anxiety 🞏

1. Compared to your current or most recent oral antipsychotic medication, which of the following would you prefer?

**Large improvement** in symptoms, but **slightly worse** digestive (gastrointestinal) problems 🞏

**Small improvement** in symptoms, but **no change** in digestive (gastrointestinal) problems 🞏

**No change** in symptoms, but **small improvement** in digestive (gastrointestinal) problems 🞏

**Slightly worse** symptoms, but **large improvement** in digestive (gastrointestinal) problems 🞏

1. Compared to your current or most recent oral antipsychotic medication, which of the following would you prefer?

**Large improvement** in symptoms, but **slightly worse** dizziness/fainting 🞏

**Small improvement** in symptoms, but **no change** in dizziness/fainting 🞏

**No change** in symptoms, but **small improvement** in dizziness/fainting 🞏

**Slightly worse** symptoms, but **large improvement** in dizziness/fainting 🞏

1. Compared to your current or most recent oral antipsychotic medication, which of the following would you prefer?

**Large improvement** in symptoms, but **slightly worse** dry mouth 🞏

**Small improvement** in symptoms, but **no change** in dry mouth 🞏

**No change** in symptoms, but **small improvement** in dry mouth 🞏

**Slightly worse** symptoms, but **large improvement** in dry mouth 🞏

1. Compared to your current or most recent oral antipsychotic medication, which of the following would you prefer?

**Large improvement** in symptoms, but **slightly worse** problems with feeling a lack of emotion 🞏

**Small improvement** in symptoms, but **no change** in problems with feeling a lack of emotion 🞏

**No change** in symptoms, but **small improvement** in problems with feeling a lack of emotion 🞏

**Slightly worse** symptoms, but **large improvement** in problems with feeling a lack of emotion 🞏

1. Compared to your current or most recent oral antipsychotic medication, which of the following would you prefer?

**Large improvement** in symptoms, but **slightly worse** drowsiness/tiredness 🞏

**Small improvement** in symptoms, but **no change** in drowsiness/tiredness 🞏

**No change** in symptoms, but **small improvement** in drowsiness/tiredness 🞏

**Slightly worse** symptoms, but **large improvement** in drowsiness/tiredness 🞏

1. Compared to your current or most recent oral antipsychotic medication, which of the following would you prefer?

**Large improvement** in symptoms, but **slightly worse** “zombie”-like feelings 🞏

**Small improvement** in symptoms, but **no change** in “zombie”-like feelings 🞏

**No change** in symptoms, but **small improvement** in “zombie”-like feelings 🞏

**Slightly worse** symptoms, but **large improvement** in “zombie”-like feelings 🞏

1. Compared to your current or most recent oral antipsychotic medication, which of the following would you prefer?

**Large improvement** in symptoms, but **slightly worse** involuntary spasms, movements, twitching, or stiffness 🞏

**Small improvement** in symptoms, but **no change** in involuntary spasms, movements, twitching, or stiffness 🞏

**No change** in symptoms, but **small improvement** in involuntary spasms, movements, twitching, or stiffness 🞏

**Slightly worse** symptoms, but **large improvement** in involuntary spasms, movements, twitching, or stiffness 🞏

1. Compared to your current or most recent oral antipsychotic medication, which of the following would you prefer?

**Large improvement** in symptoms, but **slightly worse** restlessness 🞏

**Small improvement** in symptoms, but **no change** in restlessness 🞏

**No change** in symptoms, but **small improvement** in restlessness 🞏

**Slightly worse** symptoms, but **large improvement** in restlessness 🞏

1. Compared to your current or most recent oral antipsychotic medication, which of the following would you prefer?

**Large improvement** in symptoms, but **slightly worse** sexual dysfunction 🞏

**Small improvement** in symptoms, but **no change** in sexual dysfunction 🞏

**No change** in symptoms, but **small improvement** in sexual dysfunction 🞏

**Slightly worse** symptoms, but **large improvement** in sexual dysfunction 🞏

1. Compared to your current or most recent oral antipsychotic medication, which of the following would you prefer?

**Large improvement** in symptoms, but **slightly worse** concentration problems 🞏

**Small improvement** in symptoms, but **no change** in concentratation problems 🞏

**No change** in symptoms, but **small improvement** in concentratation problems 🞏

**Slightly worse** symptoms, but **large improvement** in concentratation problems 🞏

1. Compared to your current or most recent oral antipsychotic medication, which of the following would you prefer?

**Large improvement** in symptoms, but **slightly worse** weight gain 🞏

**Small improvement** in symptoms, but **no change** in weight gain 🞏

**No change** in symptoms, but **small improvement** in weight gain 🞏

**Slightly worse** symptoms, but **large improvement** in weight gain 🞏

1. Compared to your current or most recent oral antipsychotic medication, which of the following would you prefer?

**Large improvement** in symptoms, but **slightly worse** <*other*> 🞏

**Small improvement** in symptoms, but **no change** in <*other*> 🞏

**No change** in symptoms, but **small improvement** in <*other*> 🞏

**Slightly worse** symptoms, but **large improvement** in <*other*> 🞏

**Employment**

1. What is your current employment status?

Employed (working for pay) full-time 🞏

Employed (working for pay) part-time 🞏

Unemployed and looking for work 🞏

Unemployed and not looking for work 🞏

Retired 🞏

Short-term disability 🞏

Long-term disability 🞏

Homemaker 🞏

Student 🞏

Other ________________________ 🞏

**The next several questions are about your employment status and history.**

1. Have bipolar disorder symptoms impacted your relationship with co-workers in a negative way?

Yes 🞏

No 🞏

1. Have bipolar disorder symptoms had a negative impact on your job performance?

Yes 🞏

No 🞏

1. Have your oral antipsychotic medication side effects impacted your relationship with co-workers in a negative way?

Yes 🞏

No 🞏

1. Have your oral antipsychotic medication side effects had a negative impact on your job performance?

Yes 🞏

No 🞏

1. What was your employment status when you first started having symptoms of bipolar disorder?

Employed full-time 🞏

Employed part-time 🞏

Unemployed and looking for work 🞏

Unemployed and not looking for work 🞏

Retired 🞏

Disability 🞏

Homemaker 🞏

Student 🞏

Other ________________________ 🞏

1. Have you ever changed or limited your employment status in any of the following ways because of the impact of bipolar disorder or its treatments? (Select all that apply.)

Have not changed or limited employment status 🞏

Taken leaves of absence 🞏

Reduced or changed work hours 🞏

Taken early retirement 🞏

Changed job responsibilities 🞏

Changed workplace or work environment 🞏

Taken short-term disability 🞏

Taken long-term disability 🞏

Left/Quit a job 🞏

Fired from a job 🞏

Other _____________________________ 🞏

**Demographics**

1. Which of the following best describes you? (Select all that apply.)

African-American or Black 🞏

Asian 🞏

Caucasian or White 🞏

American Indian or Alaskan Native 🞏

Hawaiian Native or other Pacific Islander 🞏

Hispanic/Spanish/or Latino origin 🞏

Other, or prefer not to answer this question 🞏

1. What is your highest degree or level of school completed as of today?

Less than high school diploma 🞏

High school diploma or GED 🞏

Some college, but no degree 🞏

Associate’s degree or Technical Certificate 🞏

Bachelor’s degree (B.A., B.S., etc.) 🞏

Graduate degree (M.A., M.S., Ph.D., M.D., etc.) 🞏
