# Supplementary material for: Antipsychotic treatment experiences of people with bipolar I disorder: patient perspectives from an online survey
Source: BMC Psychiatry. 2020 Jul 10;20:354. doi: 10.1186/s12888-020-02767-x (PMC7371473; doi:10.1186/s12888-020-02767-x)
Supplement: Supplementary file 2 — Additional file 2. Supplementary tables and figures. [file 12888_2020_2767_MOESM2_ESM.docx]

Supplementary Tables and Figures

Figure S1. Flowchart of Screening and Eligibility


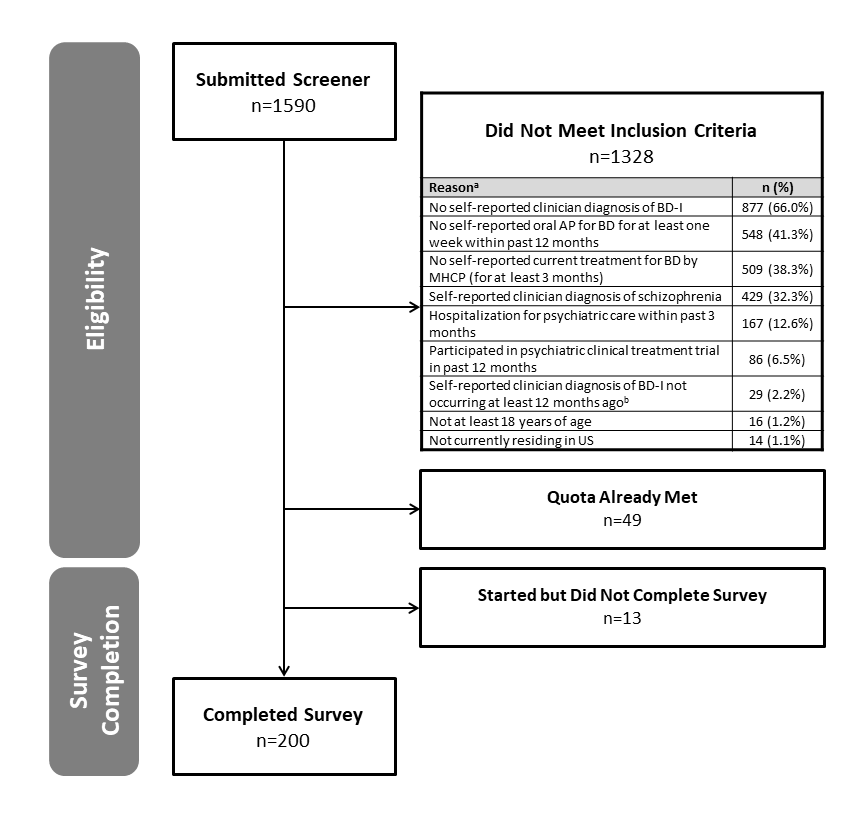


^a^ Multiple criteria may apply to each potential participant, total percentage >100%

^b^ Only potential participants that self-reported a clinician diagnosis of BD-I were administered this item

BD = Bipolar disorder; BD-I = Bipolar I disorder; MHCP = Mental health care professional; US = United States

Table S1. Symptoms Experienced During Most Recent Depressive Episode (N=200)

| **Symptom** | **n, %** |
| --- | --- |
| Decreased pleasure or interest in activities | 158 (79.0%) |
| Low mood, such as feeling sad or empty | 152 (76.0%) |
| Feeling tired or loss of energy | 150 (75.0%) |
| Anxiety | 149 (74.5%) |
| Avoiding interactions with friends, family, or co-workers | 147 (73.5%) |
| Decreased sleep or sleeping too much | 138 (69.0%) |
| Difficulty thinking, concentrating, or making decisions | 122 (61.0%) |
| Feeling worthless or guilty | 121 (60.5%) |
| Changes in weight or appetite (increased or decreased) | 116 (58.0%) |
| Feeling that life is meaningless | 101 (50.5%) |
| Loss of sex drive | 84 (42.0%) |
| Slowing down of thoughts or movement | 78 (39.0%) |
| Thoughts of your own death or a suicide attempt | 54 (27.0%) |
| Other | 1 (0.5%) |

Participants were allowed to choose more than one symptom, so the sum of percentages exceeds 100%.

Symptoms are listed in order of descending frequency.

Table S2. Symptoms Experienced During Most Recent Manic Episode (N=200)

| **Symptom** | **n (%)** |
| --- | --- |
| Anxiety | 169 (84.5%) |
| Decreased sleep | 153 (76.5%) |
| Feeling restless | 152 (76.0%) |
| Racing thoughts | 143 (71.5%) |
| Feeling angry or irritated with friends, family, or co-workers | 140 (70.0%) |
| Difficulty paying attention to one thing | 139 (69.5%) |
| Increased or faster talking | 106 (53.0%) |
| Risky or impulsive behaviors, such as spending money you don’t have or making major life changes without much thought | 87 (43.5%) |
| Extreme focus on a specific task, even to the point of ignoring other important responsibilities | 80 (40.0%) |
| High mood, such as extreme joy or excitement | 77 (38.5%) |
| Feeling that others are out to get you | 65 (32.5%) |
| Extremely high sex drive or engaging in sexual behaviors that are unusual for you | 53 (26.5%) |
| Drug/alcohol use | 48 (24.0%) |
| Thoughts of your own death or a suicide attempt | 47 (23.5%) |
| Feeling more important or powerful with respect to others than you have in the past | 37 (18.5%) |
| Visual hallucinations | 16 (8.0%) |
| Other | 3 (1.5%) |

*Participants were allowed to choose more than one symptom, so the sum of percentages exceeds 100%.

Symptoms are listed in order of descending frequency.
